# Draft Genome Sequence of the Arsenite-Oxidizing Strain *Aliihoeflea* sp. 2WW, Isolated from Arsenic-Contaminated Groundwater

**DOI:** 10.1128/genomeA.01072-13

**Published:** 2013-12-19

**Authors:** Lucia Cavalca, Anna Corsini, Vincenza Andreoni, Gerard Muyzer

**Affiliations:** Dipartimento di Scienze per gli Alimenti, la Nutrizione e l’Ambiente (DEFENS), Università degli Studi di Milano, Milan, Italya; Institute for Biodiversity and Ecosystem Dynamics, University of Amsterdam, Amsterdam, The Netherlandsb

## Abstract

Here, we report the draft genome sequence of the arsenite-oxidizing bacterium *Aliihoeflea* sp. strain 2WW, which consists of a 4.15-Mb chromosome and contains different genes that are involved in arsenic transformations.

## GENOME ANNOUNCEMENT

*Aliihoeflea* sp. strain 2WW was isolated from a biofilter treating arsenic-contaminated groundwater in the city of Cremona (Lombardia, Italy). It is a strictly aerobic, mobile, and rod-shaped Gram-negative bacterium. Phylogenetic analysis of the 16S rRNA gene sequence showed that the strain is closely (98.7%) affiliated with *Aliihoeflea aestuarii* N8^T^ (1) within the family *Phyllobacteriaceae* in the class *Alphaproteobacteria*. Strain 2WW is highly arsenic resistant, with MIC values of >200 mM and 5 mM for arsenate [As(V)] and arsenite [As(III)], respectively. Under heterotrophic conditions, it can oxidize As(III) to As(V), with specific growth rates (h^-1^) of 0.23, 0.13, and 0.006 at 30°C, 15°C, and 5°C, respectively. In growth experiments, As(III) was always oxidized to As(V) in the early exponential growth phase and the oxidation was complete within 24 h at 30°C, 96 h at 15°C, and 350 h at 5°C. Complete As(III) oxidation occurred in the pH range 5.0 to 8.0. The resting cells of an As(III)-induced culture of strain 2WW were able to oxidize completely 200 µg liter^-1^ of As(III) in 8 h, while noninduced cells oxidized As(III) in 24 h.

The genome sequence of *Aliihoeflea* sp. strain 2WW was obtained by Illumina sequencing (Illumina, Inc., San Diego, CA), producing paired-end and mate-pair sequence reads of ~50 bp, with insert sizes of ~300 bp and ~4,000 bp, respectively. CLC Genomics Workbench version 5.5.1 (CLC bio, Aarhus, Denmark) was used to assemble the quality-filtered paired-end reads into 273 contigs. Subsequently, SSPACE Premium scaffolder version 2.0 (2) and GapFiller version 1.11 (3) were used to place the contigs into 10 gap-closed scaffolds with an average sequence size of 415,210 bp. This resulted in a draft genome of 4,152,101 bp and a G+C content of 64.4%. IMG/ER (4) and RAST (5) were used to annotate the draft genome. The genome contains 4,140 genes, including 65 RNA genes. It possesses two different arsenic islands, one including 4 genes of the *ars* operon for arsenic resistance [*arsR* for regulatory protein*, acr3* for arsenite efflux pump*, arsC* for arsenate reductase, and *arsH* for protein conferring high resistance to As(V)], and the second containing an arsenite oxidase, *aioBA*. Phylogenetic analysis of *aioA* confirmed the phylogenetic affiliation with the *Alphaproteobacteria* and showed close affiliation with members of the family *Phyllobacteriaceae*, although 2WW is not chemoautotrophic. Although 2WW possesses *arsC*, normally retrieved in the *ars* operon of As(V)-resistant strains, it was not able to reduce As(V) to As(III). In addition, many genes encoding putative metal (lead, cadmium, zinc, mercury, copper, and chromium) transporters and cobalt-zinc-cadmium resistance protein (CzcD) were also identified on the genome.

### Nucleotide sequence accession number.

The draft genome sequence of *Aliihoeflea* sp. strain 2WW has been deposited in GenBank under the accession no. AYOD00000000.
